# HmsC Controls *Yersinia pestis* Biofilm Formation in Response to Redox Environment

**DOI:** 10.3389/fcimb.2017.00355

**Published:** 2017-08-08

**Authors:** Gai-Xian Ren, Xiao-Peng Guo, Yi-Cheng Sun

**Affiliations:** MOH Key Laboratory of Systems Biology of Pathogens, Institute of Pathogen Biology, Chinese Academy of Medical Sciences and Peking Union Medical College Beijing, China

**Keywords:** HmsC, biofilm formation, *Yersinia pestis*, HmsD, c-di-GMP

## Abstract

*Yersinia pestis* biofilm formation, controlled by intracellular levels of the second messenger molecule cyclic diguanylate (c-di-GMP), is important for blockage-dependent plague transmission from fleas to mammals. HmsCDE is a tripartite signaling system that modulates intracellular c-di-GMP levels to regulate biofilm formation in *Y. pestis*. Previously, we found that *Y. pestis* biofilm formation is stimulated in reducing environments in an *hmsCDE*-dependent manner. However, the mechanism by which HmsCDE senses the redox state remains elusive. Using a *dsbA* mutant and the addition of Cu^2+^ to simulate reducing and oxidizing periplasmic environments, we found that HmsC protein levels are decreased and the HmsC-HmsD protein-protein interaction is weakened in a reducing environment. In addition, we revealed that intraprotein disulphide bonds are critical for HmsC since breakage lowers protein stability and diminishes the interaction with HmsD. Our results suggest that HmsC might play a major role in sensing the environmental changes.

## Introduction

*Yersinia pestis*, the causative agent of plague, is a Gram-negative bacterium that is transmitted to mammals via bites from infected fleas. Transmission of *Y. pestis* is greatly enhanced following formation of a bacterial biofilm in the proventriculus of the flea (Eisen et al., 2006, 2007; Eisen and Gage, 2009). *Y. pestis* biofilms are positively regulated by cyclic-di-GMP (c-di-GMP), a second messenger present in numerous Gram-negative bacteria. C-di-GMP is synthesized by diguanylate cyclase (DGC) enzymes and degraded by phosphodiesterase (PDE) enzymes (Ryjenkov et al., 2005; Schmidt et al., 2005; Ryan et al., 2006). The *Y. pestis* genome encodes two DGCs, HmsT and HmsD. HmsT plays a major role in biofilm formation *in vitro*, while HmsD plays a more prominent role in blocking proventricular biofilm formation in the flea (Jones et al., 1999; Kirillina et al., 2004; Bobrov et al., 2011; Sun et al., 2011).

HmsC, a periplasmic protein, represses HmsD via direct interaction (Ren et al., 2014; Bobrov et al., 2015), while HmsE, an outer membrane protein, counteracts with HmsC and activates HmsD (Bobrov et al., 2015). The *hmsCDE* operon is homologous to the *yfiBNR* operon in *Pseudomonas aeruginosa*, which has been shown to regulate biofilm-dependent phenotypes (Malone et al., 2010, 2012). A reducing environment stimulates biofilm formation in an *yfiBRNR* (*hmsCDE*)-dependent manner (Ren et al., 2016) Consistently, mutation of *dsbA*, encoding a periplasmic protein involved in creating an oxidizing environment by catalyzing disulfide bond formation, leads to increased biofilm formation in *E. coli* and *Salmonella enteric* (Anwar et al., 2014; Hufnagel et al., 2014). However, the mechanism by which the HmsCDE (YfiBRR) system senses the redox state and regulates biofilm formation is not clear.

DsbA and DsbB coordinate to generate an oxidizing environment in the periplasm (Bardwell et al., 1993). DsbB, an inner membrane protein, oxidizes DsbA, which in turn oxidizes periplasmic proteins by catalyzing disulphide bond formation (Bardwell, 1994; Nakamoto and Bardwell, 2004). The oxidant Cu^2+^ catalyzes periplasmic disulphide bond formation and rescues defects in periplasmic disulphide bond formation in *dsbA* null strains (Battistoni et al., 1999). In the present study, we used a *dsbA* mutant and the addition of Cu^2+^ to simulate reducing and oxidizing periplasmic environments, and analyzed the role of HmsCDE. We found that in a reducing environment HmsC protein levels were decreased, and the interaction between HmsC and HmsD was weakened. Further analysis showed that HmsC contains four conserved cysteines that form two pairs of disulphide bonds that are important for protein stability and interaction with HmsD. Thus, we propose that HmsC senses the redox state in the periplasm, regulates HmsD in response and thereby controls biofilm formation in *Y. pestis*.

## Materials and methods

### Experimental procedures

#### Bacterial strains and plasmids

The strains and plasmids used are listed in Table S1. The *Y. pestis* KIM6+ strain cured plasmid pCD1 was used as the wild-type strain. KIM6+ mutants were generated by inserting PCR products into the chromosome using the Red recombination system (Datsenko and Wanner, 2000; Sun et al., 2008). All strains and plasmids were verified by PCR, DNA sequencing and plasmid complementation, as appropriate.

### *In vitro* biofilm assays

Microtiter plate biofilm assays were carried out as previously described with minor modifications (Sun et al., 2011). Briefly, cells were grown in LB broth supplemented with 4 mM MgCl_2_ and 4 mM CaCl_2_ overnight at 26°C, diluted to an OD_600_ of 0.05 using the same medium or the same medium supplemented with 0.8 mM CuSO_4_, aliquoted into 96-well polystyrene plates and incubated with shaking at 200 rpm for 24 h at 26°C. Plates were then washed three times with distilled water and adhered biofilms were stained with 0.01% crystal violet for 15 min. The dye was later re-dissolved in 80% ethanol and 20% acetone, and the absorbance at 600 nm was measured. Results from four independent experiments with at least three replicates per experiment were analyzed by one-way analysis of variance (ANOVA) with Dunnett's post-test.

### Western blotting

Overnight cultures of *Y. pestis* were diluted 1,000-fold in 50 ml LB broth at room temperature for 16 h. Samples were extracted from the same amount of stationary phase cells cultured at 26°C (100 ng total protein for detection of HmsD and HmsE, 20 ng total protein for detection of HmsC), separated by 10 or 15% SDS-PAGE, transferred to a PVDF membrane, and analyzed by immunoblotting with antibodies to the HA tag (Sigma), the Flag tag (Invitrogen) or Myc (Invitrogen). Immobilon Western HRP Substrate (Millipore) was used for detection. Results were quantitated by densitometry using NIH Image J.

### Measurement of c-di-GMP levels

Intracellular c-di-GMP levels in *Y. pestis* were measured as previously described with minor modifications (Bobrov et al., 2011; Bellows et al., 2012; Ren et al., 2014). Overnight cultures of *Y. pestis* strains were diluted to an OD_600_ of ~0.1 and grown in 40 ml LB at 26°C to an OD_600_ of ~0.8. Cell pellets were collected and resuspended in 50 μl extraction buffer (40% methanol and 40% acetonitrile in 0.1 M formic acid) per 48 mg wet cell weight. Slurries were incubated for 30 min at−20°C and insoluble material was removed by centrifugation at 4°C. Supernatants were neutralized by the addition of 4 μl 15% NH_4_HCO_3_ per 100 μl sample and 10 μl was analyzed using liquid chromatography tandem mass spectrometry. Synthetic c-di-GMP was used as a standard. Samples extracted from the *hmsT*-*hmsD* double mutant were used as a negative control. Results from three independent experiments were analyzed using one-way ANOVA with Dunnett's post-test.

### *In vivo* co-purification of HmsC and MBP-HmsD^N49−155^

Co-purification experiments were carried out as previously described (Ren et al., 2014). Briefly, the *Y. pestis hmsCDE* mutant expressing P-MBP-HmsD^N49−155^ with HmsC or HmsC^C1AC2AC3AC4A^ was inoculated into 200 ml aliquots of LB medium supplemented with kanamycin (30 μg/ml) and ampicillin (100 μg/ml). Cells were grown to an OD_600_ of 0.6 and induced by addition of arabinose to a final concentration of 0.02%. After induction for 12 h at 22°C, cells were harvested by centrifugation, resuspended in 25 ml PBS supplemented with protease inhibitor cocktail and lysed by sonication. After centrifugation at 10,000 g for 30 min at 4°C, supernatants were purified by immobilized metal affinity chromatography using Ni-NTA resin. Purified proteins were collected and tested by western blotting.

## Results

### The periplasmic redox environment regulates *Y. pestis* biofilm formation in an *hmsC*-dependent manner

**Figure 1:**
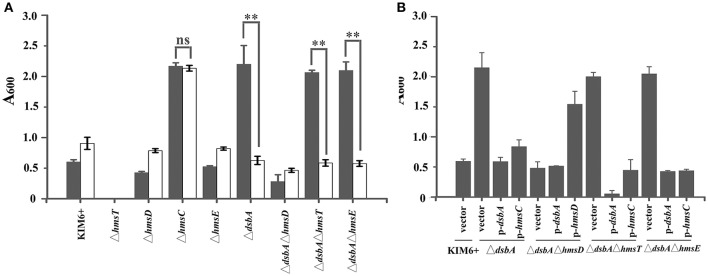
HmsC is involved in sensing the redox environment. **(A)** Relative amount of adhered biofilm formed by the *Yersinia pestis* KIM6+ parent strain and its isogenic derivatives with (gray) or without (white) CuSO_4_. **(B)** Biofilm formation by *Y. pestis* KIM6+, Δ*dsbA*, Δ*dsbA*Δ*hmsD*, Δ*dsbA*Δ*hmsT*, and Δ*dsbA*Δ*hmsE* mutant strains after transformation with the empty pUC19 vector or plasmids containing *hmsC* (pYC212), *hmsD* (pYC211) or *dsbA* (pYC257). ^**^P < 0.01, ns = not significant. Results are means and standard deviations of four independent experiments.

Previously, we found that a reducing environment stimulates biofilm formation in an HmsCDE-dependent manner (Ren et al., 2016). To explore the mechanism by which HmsCDE senses the redox state and regulates biofilm formation, we first constructed a *dsbA* null mutant to simulate a reducing environment. Biofilm formation was increased in the *dsbA* mutant strain in an HmsD-dependent manner (Figure 1A). In addition, biofilm formation also increased in the *dsbB* mutant (Figure S1). Next, we added the oxidant CuSO_4_ to simulate an oxidizing environment. Addition of CuSO_4_ significantly repressed biofilm formation in the *dsbA* mutant but not in the wild-type strain (Figure 1A). Finally, we found that addition of the reducing agent DTT was unable to increase *Y. pestis* biofilm formation when *dsbA* was overexpressed (Figure S2), suggesting that a reducing environment could be overcome by the periplasmic oxidizing environment. Taken together, these results suggest that a reducing periplasmic environment caused by *dsbA* mutation stimulates biofilm formation in *Y. pestis* and addition of the oxidant CuSO_4_ generates an oxidizing periplasmic environment that represses biofilm formation.

To further investigate the role of *hmsCDE* in response to the redox environment, we constructed a series of *dsbA* mutants and analyzed their biofilm formation. Compared with the super biofilm formation phenotype of the *dsbA* mutant, biofilm formation in the *dsbA-hmsD* double mutant was markedly decreased. However, in the *dsbA-hmsT* and *dsbA-hmsE* double mutant strains, biofilm formation was relatively unchanged compared with that in the *dsbA* single mutant (Figure 1A), suggesting that *hmsT* and *hmsE* are not involved in sensing the redox environment. The super biofilm formation phenotype of the *dsbA* mutant could be recovered by complementation with either *dsbA* or *hmsC*, or addition of CuSO_4_ in Figures 1A,B. However, the super biofilm formation phenotype caused by *hmsC* mutation could not be complemented by overexpression of *dsbA* or addition of CuSO_4_ (Figure 1A, Figure S3), suggesting that HmsC might act as a sensor of the redox environment. Taken together, these results suggest that the redox periplasmic environment regulates *Y. pestis* biofilm formation in an *hmsCDE*-dependent manner and HmsC might play an important role in sensing the redox state.

**Figure 2:**
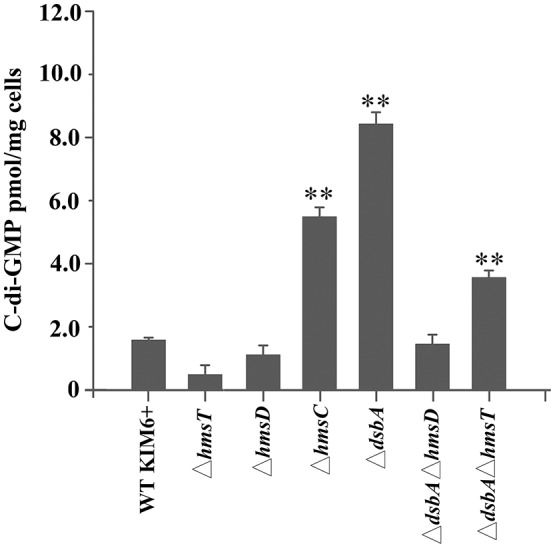
Intracellular c-di-GMP levels in *Y. pestis* and its derivatives. Intracellular c-di-GMP was extracted and measured as described in the experimental procedures. ^**^P < 0.01. Results are means and standard deviations of three independent experiments.

To further verify whether the periplasmic redox environment affects the synthesis of c-di-GMP *in vivo*, we assessed intracellular c-di-GMP levels in the different *Y. pestis* mutants. Consistent with its biofilm phenotype, the *dsbA* mutant exhibited significantly increased cellular c-di-GMP levels compared with the parent strain (Figure 2). In the absence of *dsbA*, we observed a significant increase in cellular c-di-GMP in the *hmsT* mutant but not in the *hmsD* mutant (Figure 2). Collectively, the above results suggest that HmsC might play an important role in sensing the redox state, and thereby regulate HmsD and control c-di-GMP levels, in turn regulating biofilm formation in *Y. pestis*.

### The periplasmic redox environment regulates HmsC protein levels and the interaction between HmsC and HmsD

**Figure 3:**
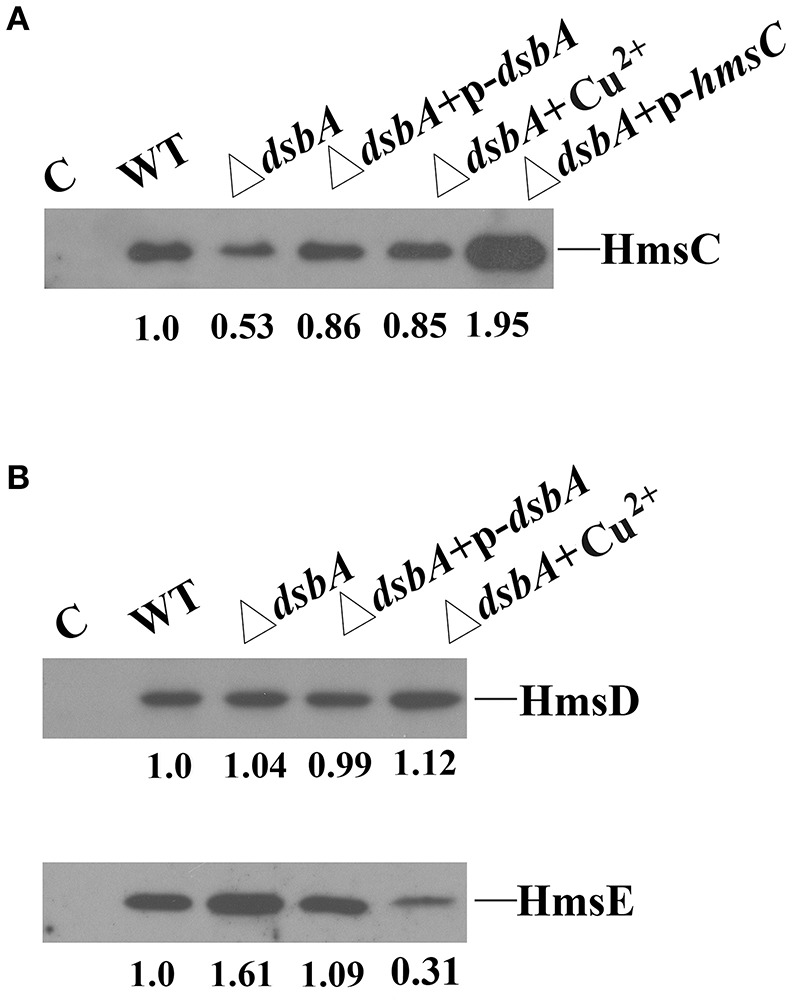
Effect of the *dsbA* mutant on HmsC, HmsD, and HmsE protein levels in *Y. pestis*. **(A)** HmsC protein levels detected by western blotting. Whole cells were extracted from *Y. pestis* SY1540 (*hmsC*-Flag) and *Y. pestis* SY1542 (Δ*dsbA, hmsC*-Flag) containing plasmid pYC257 (p-*dsbA*) or pYC212 (p-*hmsC*), and from cells grown in LB medium supplemented with CuSO_4_. **(B)** HmsD and HmsE protein levels detected by western blotting. Whole cells were extracted from *Y. pestis* SY1562 (*hmsD*-Myc), SY2046 (*hmsE*-HA), *Y. pestis* SY1564 (Δ*dsbA, hmsD*-Myc), and *Y. pestis* SY2994 (Δ*dsbA, hmsE*-HA) containing plasmid pYC257 (p-*dsbA*), and from cells supplemented with CuSO_4_. HmsC, HmsD, and HmsE protein levels were quantified using Image J. Numbers below blots indicate the ratio of proteins in the indicated sample compared with samples collected at mid-stationary phase (Set as 1) based on at least two independent experiments.

**Figure 4:**
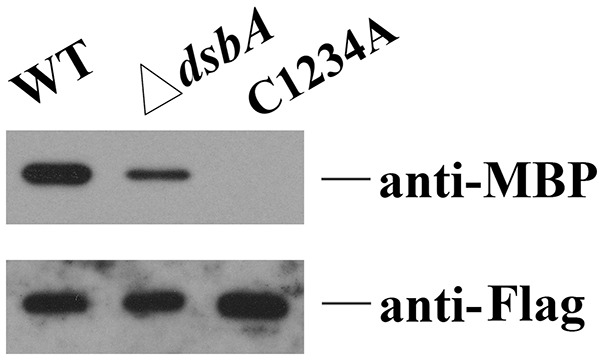
Disulphide bond formation affects the interaction between HmsC and HmsD. HmsC, and HmsC mutants were co-purified with the periplasmic domain of HmsD *in vivo*. P-MBP-HmsD^N49−155^ was co-expressed with HmsC-His_8_ in the wild-type strain (lane 1) or the dsbA mutant (lane 2). P-MBP-HmsD^N49−155^ was co-expressed with HmsC^C87AC126AC161AC168A^-Flag-His_8_(C1234A) (lane 3), purified using Ni-NTA resin, and detected with anti-MBP tag and anti-Flag antibodies.

The redox environment might affect the expression of HmsCDE or the regulatory role of HmsC on HmsD to modulate biofilm formation. To test this hypothesis, we first analyzed HmsCDE protein levels under different redox conditions. HmsD protein levels were not affected by mutation of *dsbA* (Figure 3B), while HmsC was slightly decreased and HmsE was slightly increased in the *dsbA* mutant (Figure 3). Meanwhile, addition of the oxidant CuSO_4_ stabilized HmsC in the *dsbA* mutant (Figure 3A). These results suggest that the protein level of HmsC is affected by the periplasmic redox environment. We previously reported that HmsC has a direct interaction with the periplasmic region of HmsD and the loss of HmsC function releases the repression of HmsD, in turn stimulating biofilm formation. To investigate whether the periplasmic redox environment affects the interaction between HmsC and HmsD, we performed co-purification analysis of HmsC and the periplasmic domain of HmsD as previously described (Ren et al., 2014). As shown in Figure 4, the periplasmic domain of HmsD was co-purified with HmsC to a lesser extent in the *dsbA* mutant than in the wild-type strain, suggesting that the interaction between HmsC and HmsD is affected by the periplasmic redox environment. Taken together, these results suggest that the redox state might regulate biofilm formation through modulation of the conformation of the HmsC protein and its interaction with HmsD.

### Disulphide bond formation is critical for HmsC function

**Figure 5:**
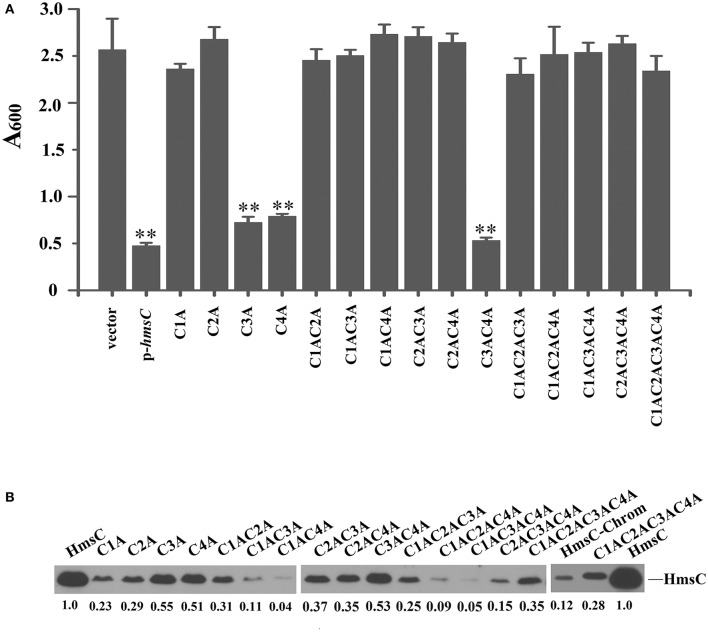
Disulphide bonds are important for HmsC function. **(A)** Relative amount of adhered biofilm from the *hmsC* mutant strain and *hmsC* mutant strains transformed with the pUC19 vector or the plasmids containing *hmsC* (pYC212), *hmsC*^C87A^ (pYC316, C1A), *hmsC*^C126A^ (pYC317, C2A), *hmsC*^C161A^ (pYC318, C3A), *hmsC*^C168A^ (pYC317, C4A), *hmsC*^C87AC126A^ (pYC320, C1AC2A), *hmsC*^C87AC161A^ (pYC321, C1AC3A), *hmsC*^C87AC168A^ (pYC322, C1AC4A), *hmsC*^C126AC161A^ (pYC323, C2AC3A), *hmsC*^C126AC168A^ (pYC324, C2AC4A), *hmsC*^C161AC168A^ (pYC325, C3AC4A), *hmsC*^C87AC126AC161A^ (pYC326, C1AC2AC3A), *hmsC*^C87AC126AC168A^ (pYC327, C1AC2AC4A) or *hmsC*^C87AC126AC161AC168A^ (pYC333, C1AC2AC3AC4A). **(B)** Protein levels in *hmsC* mutant strains transformed with the empty pUC19 vector, or plasmids containing *hmsC* (pYC212) or *hmsC* conserved cysteine mutants. ^**^P < 0.01. Results are means and standard deviations of four independent experiments. HmsC protein levels were quantified using Image J. Numbers below blots indicate the ratio of proteins in the indicated samples compared with samples collected at mid-stationary phase (Set as 1) based on at least two independent experiments.

Sequence alignment of HmsC and its homolog YfiR revealed four conserved cysteines in HmsC (Figure S4). Analysis of the crystal structure of YfiR suggests that these cysteines form two disulphide bonds: Cys71-Cys110 (corresponding to Cys87-Cys126 in HmsC) and Cys145-Cys152 (corresponding to Cys161-Cys168 in HmsC) (Yang et al., 2015). Since disulphide bond formation is dependent on the redox environment, we hypothesized that the redox state affects disulphide bond formation, and thereby regulates the conformation of HmsC and its interaction with HmsD. If so, breakage of the disulphide bonds will likely affect HmsC function, resulting in hyper biofilm formation. To test this hypothesis, we constructed a series of HmsC mutants in which the four conserved cysteine were replaced with alanine. Although the Cys145-Cys152 disulphide bond in YfiR (corresponding to Cys161-Cys168 in HmsC) is critical for protein folding (Yang et al., 2015), the function of HmsC was only partially lost when Cys161 or Cys168 was mutated (Figure 5A), whereas HmsC function was completely lost when Cys87 or Cys126 was mutated (Figure 5A). This suggests that the Cys87-Cys126 disulphide bond plays a critical role in maintaining the structure and function of HmsC.

To further determine whether breakage of disulphide bonds affects the stability of HmsC, we analyzed the expression levels of HmsC mutants. HmsC fusion proteins containing the Flag tag, which displayed a similar phenotype to mutants of HmsC without the tag (Ren et al., 2014), were constructed and expressed in the *Y. pestis hmsC* mutant. As shown in Figure 5B, breakage of the Cys161-Cys168 disulphide bond had a slight effect on HmsC protein levels, whereas breakage of the Cys87-Cys126 disulphide bond had a much more significant effect. These results suggest that disulphide bonds are critical for maintaining the structure and function of HmsC. Surprisingly, although HmsC was non-functional when the Cys87-Cys126 disulphide bond was broken, the amount of HmsC mutant protein (Cys87 and/or Cys126) expressed from the respective plasmid was higher than the amount of wild-type HmsC expressed from the chromosome (Figure 5B). This suggests that disulphide bond formation affects the role of HmsC not only by regulating the stability of the HmsC protein, but also by regulating its interaction with HmsD. To verify this hypothesis, we performed co-purification analysis of HmsC and the periplasmic domain of HmsD. Consistent with the previous results that *dsbA* mutation affect the co-purification of HmsC and HmsD (Figure 4), HmsC mutant (C1234A) lacking disulphide bonds could not be co-purified with HmsD, suggesting that disulphide bond formation is crucial for the interaction between HmsC and HmsD.

## Discussion

Bacteria are exposed to a constantly changing environment, and they are required to sense and react to these changes in order to adapt and thrive. HmsCDE and its homolog YfiBNR are involved in sensing environmental conditions and regulating biofilm formation (Malone et al., 2010, 2012; Ren et al., 2014; Bobrov et al., 2015). In response to cell stress, the outer membrane protein YfiB/HmsE sequesters the periplasmic protein YfiR/HmsC, alleviating its inhibition of YfiN/HmsD in the inner membrane, and thus provoking the diguanylate cyclase activity of YfiN to induce c-di-GMP production (Malone et al., 2012; Bobrov et al., 2015). However, HmsE is not required for increased biofilm formation in a reducing environment (Ren et al., 2016). In addition, *yfiB* is absent in some bacteria such as *Salmonella* and *Dickeya* (Malone et al., 2010, 2012), which contain fully conserved *yfiR* and *yfiN*, suggesting that there might be another protein involved in sensing environmental signals. Herein, we report that the periplasmic protein HmsC plays an important role in sensing the redox state of the environment and regulation of biofilm formation.

The crystal structure of YfiR (Li et al., 2015; Yang et al., 2015), a homolog of HmsC, revealed two pairs of intraprotein disulphide bonds, Cys71-Cys110 and Cys145-Cys152, which are conserved in homologs in many bacteria (Figure S4). The disulphide bonds are important for the stability of HmsC and the interaction of HmsC-HmsD (Figures 4, 5B). In addition, we found that the Cys87-Cys126 disulphide bond in HmsC (corresponding to Cys71-Cys110 in YfiR) might play a major role in sensing the redox state and regulate the function and stability of HmsC. This disulphide bond can only form in oxidative conditions (Yang et al., 2015) and was found to be critical for the function of HmsC (Figure 5). By contrast, the Cys145-Cys152 disulphide bond in YfiR remained intact in the reducing environment of the cytoplasm (Yang et al., 2015) and breakage of the corresponding disulphide in HmsC only partially affected its function (Figure 5A).

**Figure 6:**
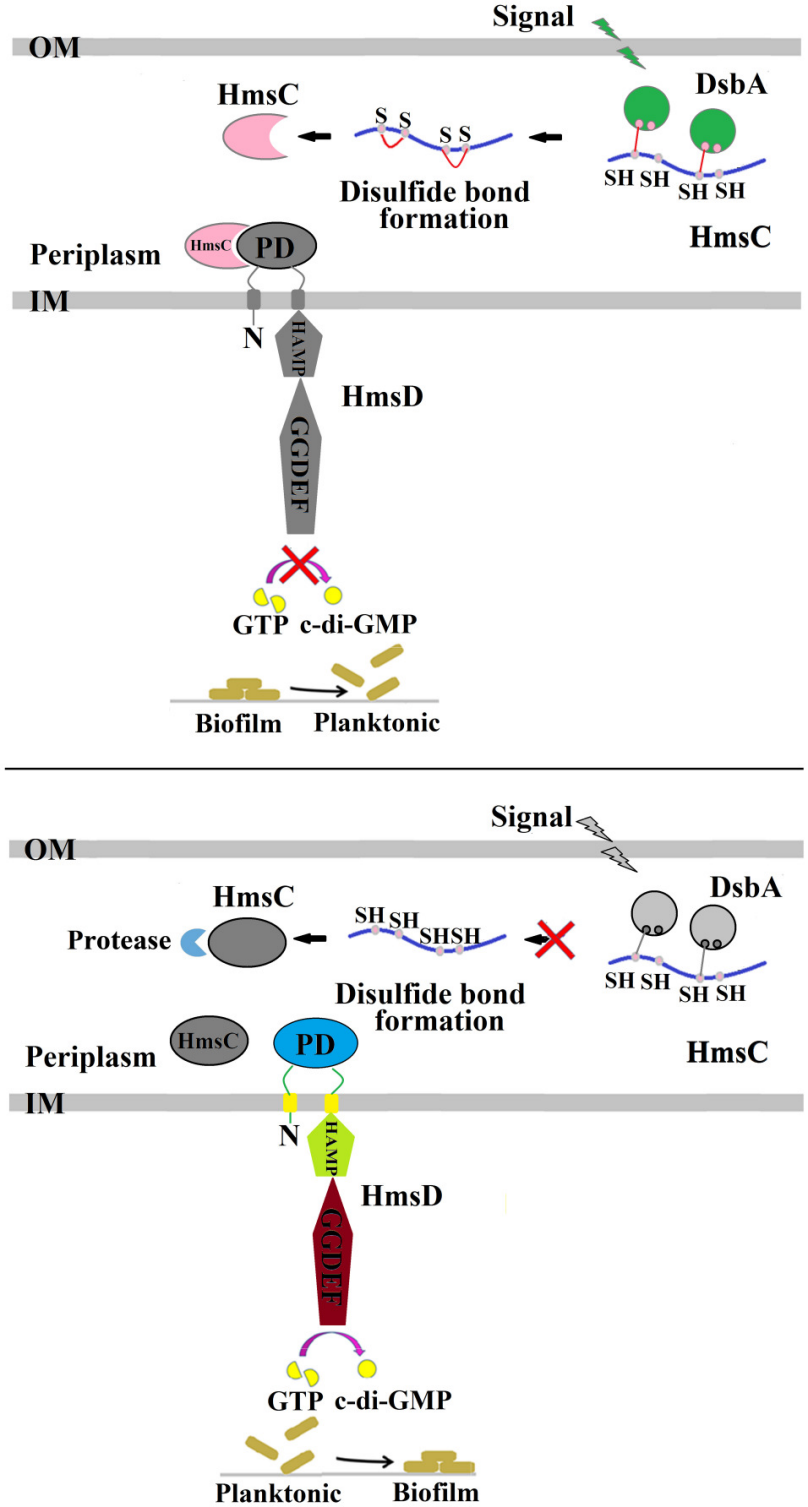
Regulation of *Y. pestis* biofilm formation by DsbA and HmsC. The periplasmic protein HmsC possesses four conserved cysteines that likely form disulphide bonds that are modulated by DsbA. The formation of disulphide bonds controls HmsC protein degradation and protein conformation, and determines whether HmsC binds to or disassociates from the periplasmic sensor domain of HmsD, which controls the activity of HmsD that in turn regulates the intracellular c-di-GMP levels and ultimately biofilm formation.

The formation of disulphide bonds in HmsC might be modulated in response to changes in the redox environment in the periplasm, which in turn could regulate the stability of the HmsC protein and its interaction with HmsD. Further modulation via HmsC inhibition might regulate the diguanylate cyclase activity of HmsD and thus control biofilm formation (Figure 6). The redox state in the periplasm can be affected by substances such as DTT and Cu^2+^ that generate reducing and oxidizing conditions, respectively. However, the periplasmic redox environment is mainly controlled by the DsbA/B system, which coordinates disulphide bond formation in periplasmic proteins. Thus, environmental signals could modulate the DsbA/B system, which in turn regulates HmsC and controls biofilm formation.

## Author contributions

Experiment designation: GR and YS; Experiment carry out: GR and XG; Manuscript writing: GR and YS; Manuscript review and modification: GR and YS.

### Conflict of interest statement

The authors declare that the research was conducted in the absence of any commercial or financial relationships that could be construed as a potential conflict of interest.
