# Corrigendum: HmsC Controls *Yersinia pestis* Biofilm Formation in Response to Redox Environment

**DOI:** 10.3389/fcimb.2017.00525

**Published:** 2018-01-12

**Authors:** Gai-Xian Ren, Xiao-Peng Guo, Yi-Cheng Sun

**Affiliations:** MOH Key Laboratory of Systems Biology of Pathogens, Institute of Pathogen Biology, Chinese Academy of Medical Sciences and Peking Union Medical College, Beijing, China

**Keywords:** HmsC, biofilm formation, *Yersinia pestis*, HmsD, c-di-GMP

In the original article, the order of the funders in the funding statement was incorrect. It should read:

This work was supported by the National Major Research & Development Program of China (2016YFC1202600), the National Basic Research Program of China (973 Program) (2015CB554200), the National Natural Science Foundation of China (81501723) and (31670139) and CAMS Initiative for Innovative Medicine (2016-I2M-1-013).

The authors apologize for this error and state that it does not affect the conclusions of the article in any way.

The original article has been updated.

## Conflict of interest statement

The authors declare that the research was conducted in the absence of any commercial or financial relationships that could be construed as a potential conflict of interest.

