# Supplementary material for: HmsC Controls Yersinia pestis Biofilm Formation in Response to Redox Environment
Source: Front Cell Infect Microbiol. 2017 Aug 8;7:355. doi: 10.3389/fcimb.2017.00355 (PMC5550408; doi:10.3389/fcimb.2017.00355)
Supplement: Supplementary file 1 [file DataSheet1.DOC]

| TABLE S1. Strains and plasmids used in this study | | | |
| --- | --- | --- | --- |
| Strain or plasmid | Genotype and/or description | Reference or source | |
| *Y. pestis* |  | |  |
| KIM6+  CDY362  CDY469  CDY497  SY100  SY879  SY882  SY1055  SY1314  SY1334  SY1336  SY1540  SY1542  SY1562  SY1564  SY2046  Plasmid  pYC212  PYC257  PYC316  PYC317  PYC318  PYC319  PYC320  PYC321  PYC322  PYC323  PYC324  PYC325  PYC326  PYC327  PYC323 | wild type (pCD1-)  Δ*hmsP::Kan*  Δ*hmsD::Kan*  Δ*hmsT::Cm*  Δ*hmsC::Kan*  Δ*dsbA::Kan*  Δ*hmsT::Cm*Δ*dsbA::Kan*  Δ*hmsD*Δ*dsbA::Kan*  Δ*dsbB::Kan*  Δ*dsbC::Kan*  Δ*dsbD::Kan*  Chromosomal *hmsC-*Flag  Δ*dsbA::Kan* Chromosomal *hmsC-*Flag  Chromosomal *hmsD-*MYC  Δ*dsbA::Kan* Chromosomal*hmsD-*MYC  Chromosomal *hmsE-*HA  hmsCexpressed in pUC19  *dsbA* expressed in pUC19  *hmsC*^C87A^*-*FLAG in pUC19  *hmsC*^C126A^*-*FLAG in pUC19  *hmsC*^C161A^*-*FLAG in pUC19  *hmsC*^C168A^*-*FLAG in pUC19  *hmsC*^C87AC126A^*-*FLAG in pUC19  *hmsC*^C87AC161A^*-*FLAG in pUC19  *hmsC*^C87AC168A^*-*FLAG in pUC19  *hmsC*^C126AC161A^*-*FLAG in pUC19  *hmsC*^C126AC168A^*-*FLAG in pUC19  *hmsC*^C161AC168A^*-*FLAG in pUC19  *hmsC*^C87AC126AC161A^*-*FLAG in pUC19  *hmsC*^C87AC126AC168A^*-*FLAG in pUC19  *hmsC*^C87AC126AC161AC168A^*-*FLAG in pUC19 | | (Deng et al., 2002)  ([Sun et al., 2011](#_ENREF_26))  ([Sun et al., 2011](#_ENREF_26))  ([Sun et al., 2011](#_ENREF_26))  ([Sun et al., 2011](#_ENREF_26))  This study  This study  This study  This study  This study  This study    This study  This study  This study  This study  This study  This study  This study  This study  This study  This study  This study  This study  This study  This study  This study  This study |
| PYC903  PYC904 | *hmsD*^N49-155^expressed in pMal-p5x  *hmsD*^N49-155^expressed in pMal-c5x | |  |

Deng, W., Burland, V., Plunkett, G. III, Boutin, A., Mayhew, G. F., Liss, P., et al. (2002). Genome sequence of *Yersinia pestis* KIM. J. Bacteriol. 184, 4601–4611. doi: 10.1128/JB.184.16.4601-4611.2002.

Sun, Y.C., Koumoutsi, A., Jarrett, C., Lawrence, K., Gherardini, F.C., Darby, C., et al. (2011). Differential control of Yersinia pestis biofilm formation in vitro and in the flea vector by two c-di-GMP diguanylate cyclases. PLoS One 6(4), e19267. doi: 10.1371/journal.pone.0019267 PONE-D-10-06594 [pii].

Ren, G.X., Yan, H.Q., Zhu, H., Guo, X.P., and Sun, Y.C. (2014). HmsC, a periplasmic protein, controls biofilm formation via repression of HmsD, a diguanylate cyclase in Yersinia pestis. *Environ Microbiol* 16(4)**,** 1202-1216. doi: 10.1111/1462-2920.12323.

Ren, G.X., Fan, S., Guo, X.P., Chen, S., and Sun, Y.C. (2016). Differential Regulation of c-di-GMP Metabolic Enzymes by Environmental Signals Modulates Biofilm Formation in Yersinia pestis. *Front Microbiol* 7**,** 821. doi: 10.3389/fmicb.2016.00821.


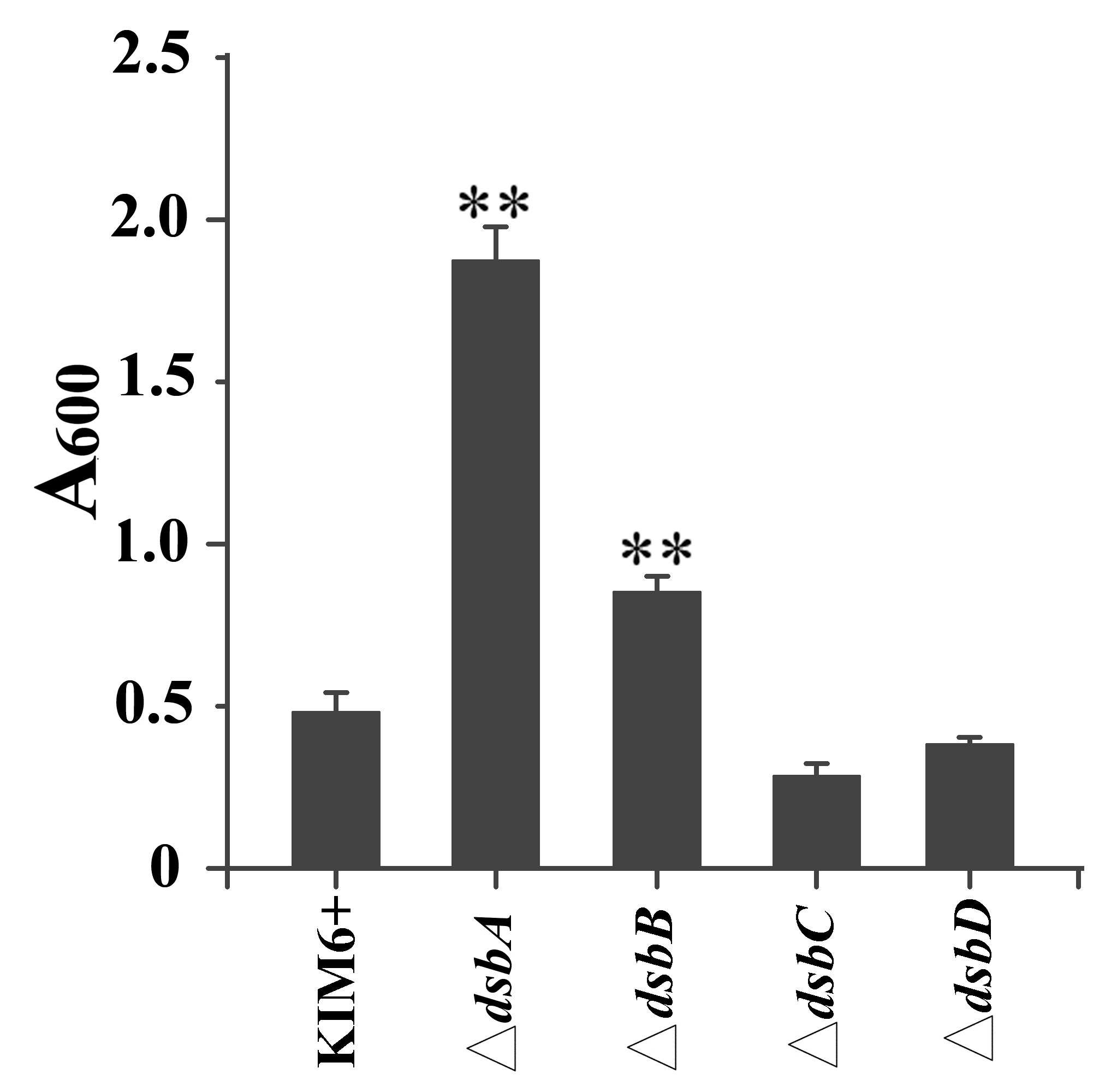


**Figure S1. Effect of *dsb* mutation on *Y. pestis* biofilm formation.** Relative amounts of adherent biofilm made by the *Y. pestis* KIM6+ parent strain and its isogenic derivatives. **P<0.01. The mean and standard deviation of four independent experiments are indicated.


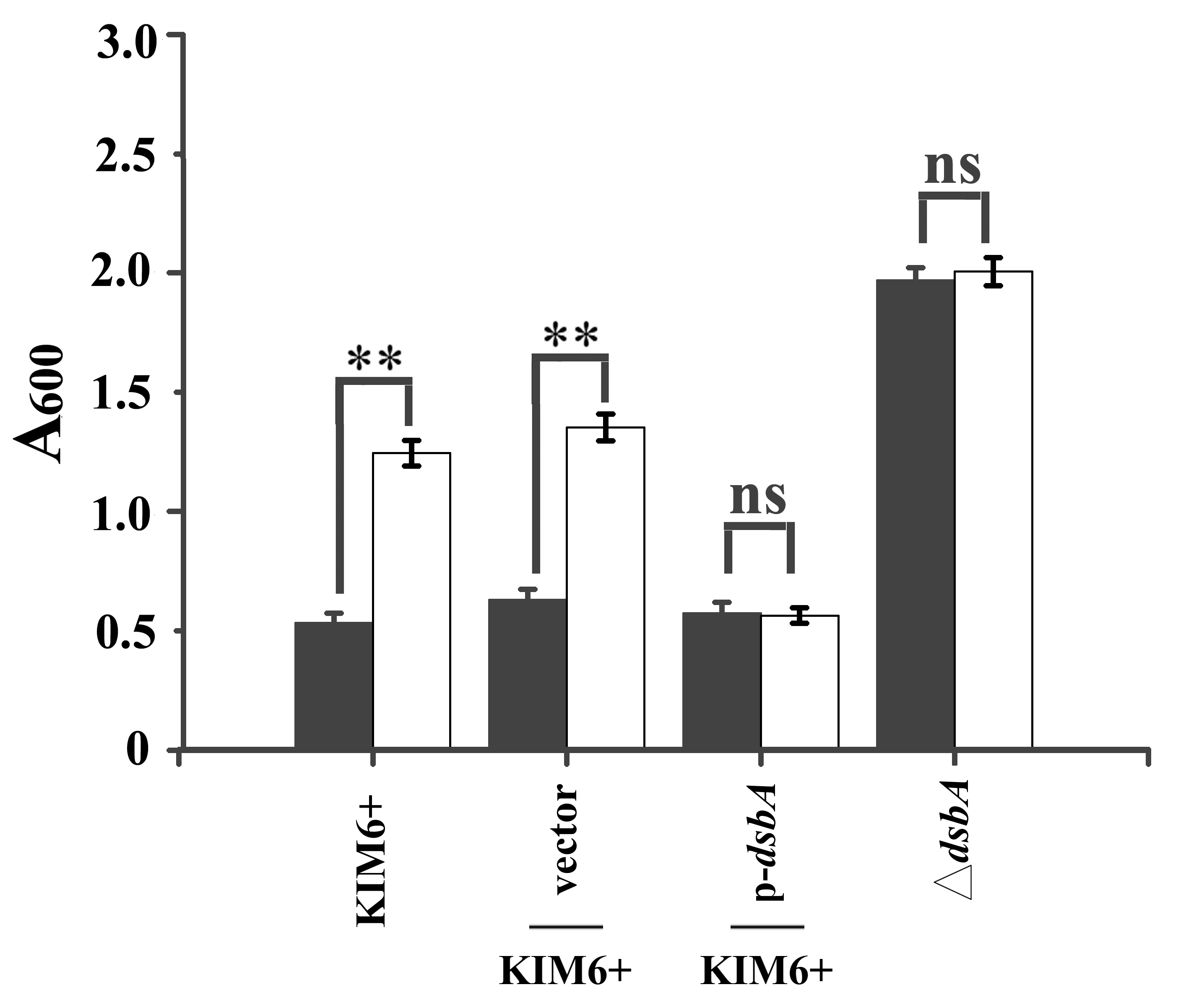


**Figure S2. Effect of DTT on *Y. pestis* biofilm formation *in vitro*.** Relative amounts of adherent biofilm formed by the *Y. pestis* KIM6+ parental strain, the *dsbA* mutant and *Y. pestis* KIM6+ parental strain after transformation with the empty pUC19 vector or plasmids containing *dsbA* (pYC257). The culture was supplemented with (white bars) or without (grey bars) 4mM DTT. **P<0.01. ns, not significant. The mean and standard deviation of three independent experiments are indicated.


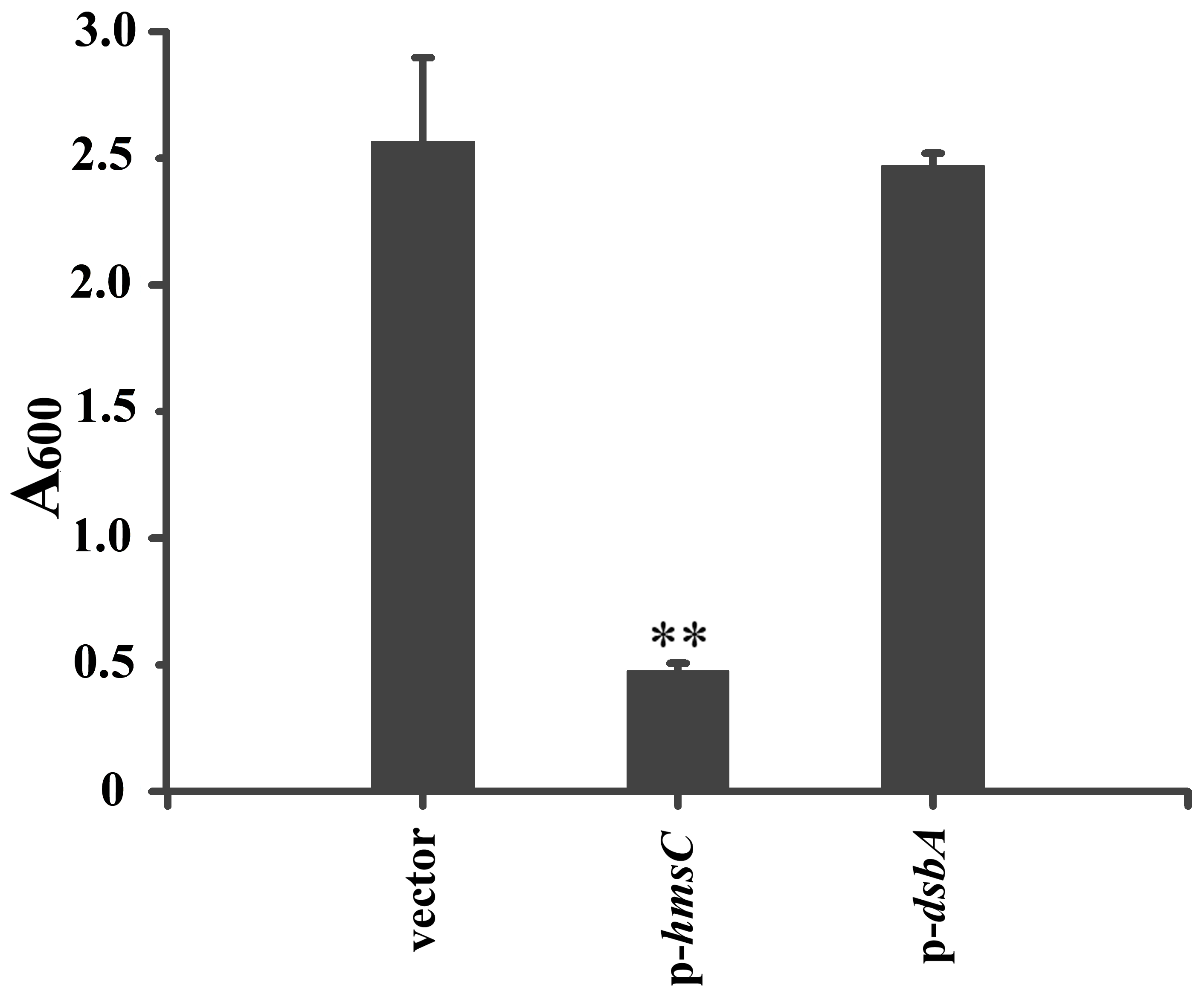


**Figure S3. Effect of overexpression of *dsbA* on *Y. pestis* biofilm formation.** Relative amounts of adherent biofilm made by the *Y. pestis* Δ*hmsC* mutant strain and Δ*hmsC* mutant strains after transformation with the empty pUC19 vector or plasmids containing *hmsC* (pYC212), *dsbA* (pYC257).**P<0.01. The mean and standard deviation of four independent experiments are indicated.


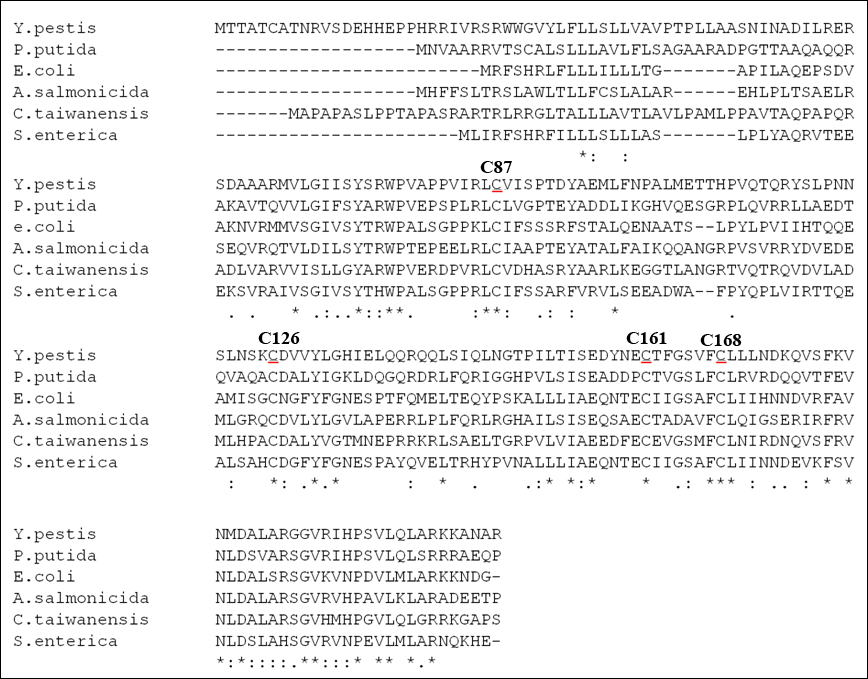


**Figure S4. Sequence alignment analysis of *Y. pestis* HmsC with its homologs**. Multiple sequence alignment performed using ClustalW. Asterisks, identical residues; periods, conserved residues; colons, strongly conserved residues. The homologs of HmsC shown are from Escherichia coli K-12, Salmonella Typhimurium LT2, Pseudomonas aeruginosa PAO1, Pseudomonas putida KT2440, Cupriavidus taiwanensis LMG 19424 and Aeromonas salmonicida A449.
